# The Back 2 Activity Trial: education and advice versus education and advice plus a structured walking programme for chronic low back pain

**DOI:** 10.1186/1471-2474-11-163

**Published:** 2010-07-15

**Authors:** Suzanne M McDonough, Mark A Tully, Seán R O'Connor, Adele Boyd, Daniel P Kerr, Siobhán M O'Neill, Antony Delitto, Ian Bradbury, Catrine Tudor-Locke, David G Baxter, Deirdre A Hurley

**Affiliations:** 1 Health and Rehabilitation Sciences Research Institute, University of Ulster Shore Road, Newtownabbey, Co Antrim, BT37 0QB, UK; 2 UKCRC Centre of Excellence for Public Health (NI), Queens' University Belfast, UK; 3 School of Psychology, University of Ulster, UK; 4 School of Health and Rehabilitation Science, University of Pittsburgh, USA; 5 Frontier Science, UK; 6 Walking Behavior Laboratory, Pennington Biomedical Research Center, Baton Rouge, LA, USA; 7 Centre for Physiotherapy Research, University of Otago, New Zealand; 8 School of Public Health, Physiotherapy and Population Science, University College Dublin, Ireland

## Abstract

**Background:**

Current evidence supports the use of exercise-based treatment for chronic low back pain that encourages the patient to assume an active role in their recovery. Walking has been shown it to be an acceptable type of exercise with a low risk of injury. However, it is not known whether structured physical activity programmes are any more effective than giving advice to remain active.

**Methods/Design:**

The proposed study will test the feasibility of using a pedometer-driven walking programme, as an adjunct to a standard education and advice session in participants with chronic low back pain. Fifty adult participants will be recruited via a number of different sources. Baseline outcome measures including self reported function; objective physical activity levels; fear-avoidance beliefs and health-related quality of life will be recorded. Eligible participants will be randomly allocated under strict, double blind conditions to one of two treatments groups. Participants in group A will receive a single education and advice session with a physiotherapist based on the content of the 'Back Book'. Participants in group B will receive the same education and advice session. In addition, they will also receive a graded pedometer-driven walking programme prescribed by the physiotherapist. Follow up outcomes will be recorded by the same researcher, who will remain blinded to group allocation, at eight weeks and six months post randomisation. A qualitative exploration of participants' perception of walking will also be examined by use of focus groups at the end of the intervention. As a feasibility study, treatment effects will be represented by point estimates and confidence intervals. The assessment of participant satisfaction will be tabulated, as will adherence levels and any recorded difficulties or adverse events experienced by the participants or therapists. This information will be used to modify the planned interventions to be used in a larger randomised controlled trial.

**Discussion:**

This paper describes the rationale and design of a study which will test the feasibility of using a structured, pedometer-driven walking programme in participants with chronic low back pain.

**Trial Registration:**

[ISRCTN67030896]

## Background

Low back pain (LBP) has a high lifetime prevalence with non-specific LBP representing the large majority of cases [1]. Relapses in pain (60%) and work absences (33%) are common [1] leading it to be one of the most costly conditions in the UK (total cost of £stg10,668 million) [1]. Current research evidence supports the use of exercise-based treatment programmes for chronic LBP (CLBP pain persisting for at least 12 weeks) that encourage the patient to assume an active role in their recovery [2]. Although the European Guidelines have advocated exercise as the first line of treatment in the management of CLBP it is not clear which type of exercise works best or what the 'active ingredient' of exercise therapy is. Therefore, it has been suggested that much more research is required to allow the development of a wider range of low cost, but effective exercise programmes [1].

There is however a significant challenge in encouraging people with CLBP to become more physically active as this group often exhibit fear avoidance behaviour, resulting in decreased physical activity (PA), in the belief that this will limit exacerbations in their LBP [3]. However given the evidence for the benefit of regular exercise in people who cope with their CLBP, approaches that encourage and motivate long-term lifestyle changes in PA are required [4]. A Cochrane review of interventions that promote PA concluded that a mixture of professional guidance, self-direction and on-going professional support can encourage adults to be more physically active [5]. One type of intervention that has been shown to be effective in increasing PA and which incorporates these aspects is pedometer-driven walking [6].

Walking is an ideal intervention for physically inactive individuals. In previous research, we have shown it to be a very acceptable type of exercise [7], as it does not require training or equipment and can be undertaken in an individual's own locality and time [8], with little risk of injury in sedentary, healthy individuals [9]. Recent walking initiatives, such as Walking the Way to Health Initiative and the National Step-O-Meter campaign http://www.whi.org.uk have been advocated by the Chartered Society of Physiotherapy [10]. The Step-O-Meter campaign has focused on the recommendation that individuals should accumulate 10,000 steps per day [11], using pedometers to set goals and record compliance [12]. Pedometers are simple to use, inexpensive devices which produce a user-friendly output (steps/day). A recent meta-analysis has shown they are effective motivators to taking more activity and lead to health improvements (reduction in blood pressure and body mass index (BMI)) [13]. However, these findings need to be extended from healthy sedentary middle-aged adults to patient groups.

In the UK, anecdotal evidence suggests that people with CLBP can reduce their pain through walking, and practitioners are being encouraged to loan pedometers in order to increase PA [10]. However, there is little evidence to guide this practice. Only one published study has investigated walking (unsupervised or monitored), the outcomes of which were less effective than those following physiotherapy or exercise therapy [14] and another trial is ongoing [15]. In contrast one study (of chiropractic versus medical management) suggested that those engaged in additional unsupervised brisk walking (>3 hours per week) had a better outcome [16]. Limited conclusions can be drawn from these studies as participants did not receive a structured walking programme. Therefore, given that there are no previous trials of the use of pedometers in CLBP; research is required so that guidelines can be drawn up on how best to implement this type of programme.

It is not known whether this type of structured and tailored motivational programme is likely to be more effective than simply giving the patient advice to remain active and promoting self-management strategies via an educational booklet. Such booklets (e.g. the Back Book [17]), which are recommended by both the American and European Guidelines [18,1], have been shown to be similar or only slightly inferior in effectiveness to costlier interventions (physiotherapy, supervised exercise classes, yoga, spinal manipulation, acupuncture and massage) [17,19].

## Aim

The aim of the study is to test the feasibility of using a structured, pedometer-driven walking programme, as an adjunct to a standard education and advice session in patients with CLBP. This work will add to ongoing research in this area by some of the team [15].

### Specific objectives are as follows

**i**. To assess recruitment and adherence rates in education and advice and education and advice plus walking programme groups.

**ii**. To determine the incidence of adverse events, including musculo-skeletal injuries, and level of overall satisfaction in both groups.

**iii**. To conduct a qualitative exploration of participants' experience of the walking plus education group.

**iv**. To make between and within group comparisons and estimate effect sizes for change in functional disability, PA levels, stage of change, fear-avoidance, self-efficacy, health-related quality of life, psychosocial beliefs, general health and participant satisfaction.

## Methods/Design

### Design

Single-blinded feasibility study.

### Ethical approval

Ethical approval for the trial has been granted by The Office for Research Ethics Committees (Northern Ireland) [Ref No. 09/NIR01/49]. All patients who agree to take part will be required to give informed written consent prior to participation in the study.

### Study population

50 adult participants (Male or Female; aged 18 years or over) with LBP persisting for at least twelve weeks will be included in the study. Participants will be recruited through a number of different sources using previously employed methods [20].

### Identification of potential participants and screening procedures

In order to maximise recruitment and to ensure that the sample size is achieved, potential participants will be identified from a number of sources.

I) The Physiotherapy department at the Robinson Memorial Hospital, Ballymoney will serve as one recruitment source. Potential participants will be identified from the referral lists and sent a letter inviting them to take part in the research study. This letter will state clearly that the individual is under no obligation to take part in the study and that non-participation will not prevent them from still receiving a standard physiotherapy appointment after the 9-13 week waiting list. The 8 week intervention period of the study therefore fits within this timescale. Interested participants will contact the Physiotherapy department to arrange an initial appointment time. They will then be sent a confirmation letter and information sheet which will allow for a 'cooling off' period of approximately one week.

II) Retrospective searches of computerised records of local General Practices will be conducted by practice staff with the assistance of the Clinical Trials Practitioner (CTP) from the Northern Ireland Primary Care Research Network (PCRN). Potential participants will be sent an invitation letter and information sheet from the study team along with a covering letter from their GP. Potential participants will be asked to complete an enclosed reply slip indicating their interest in being contacted by a member of the research team with a view to taking part in the study. They will also have the opportunity to indicate that they are not interested in the study and do not wish to be contacted further using the same reply slip. Potential participants who do not return the reply slip will also be followed up by a telephone call from the CTP at least two weeks after the invitation letter was sent. No further contact will be made after this stage with individuals who indicate that they do not wish to take part. Those who do respond positively to the invitation letter or telephone call from CTP will then be contacted separately by telephone and screened for eligibility by a member of the local research team. Those meeting the inclusion criteria will then be sent a second letter with an appointment time arranged for approximately one week after telephone screening. This is in order to allow an adequate 'cooling-off' period during which the individual can fully consider their participation in the study.

III) A third potential source for recruitment will be identification of participants via Occupational Health or via email/poster advertisements to staff and students at the University of Ulster.

At the first appointment, a full verbal explanation of the study procedures will be provided and eligibility to participate confirmed using the inclusion and exclusion criteria shown in table 1. Written informed consent will then be sought from eligible participants. At this stage a final exclusion criteria will be applied. Apparently eligible participants will be fitted with an activPAL physical activity monitor (PAL Technologies, Glasgow, UK) for one week. This is in order to provide an objective measurement of PA levels and to confirm that the individual does not have a pre-existing high level of PA. This will be determined according to previously defined daily step count categories [11]. To meet the final criteria for eligibility, the individual must be taking less than an average of 8,500 steps/day. Participants meeting the criteria outlined above will then be invited back one week later to begin the intervention period of the study. Details of the recruitment, screening and study procedures are outlined in Additional file 1. Participants recruited via method I will receive the intervention in the Physiotherapy department while participants recruited via methods II and III will receive the intervention in the research centre at the University of Ulster.

**Table 1:** Inclusion and exclusion criteria

Inclusion	Exclusion
Males and females aged 18 years or over	Any spinal surgery in the past twelve months
LBP with/without radiation persisting for greater than 12 weeks.	Evidence of nerve root, spinal cord, or cauda equina compression, severe spinal stenosis indicated by signs of neurogenic claudication, Grade 3-4 spondylolisthesis, (Grade 1-2 spondylolisthesis eligible for inclusion) fibromyalgia or systemic/inflammatory disorder
Capable of participating in home based exercise as indicated by their GP [home based, walking intervention]	Any other current musculoskeletal injury or contraindication to increasing physical activity levels, including any cardio-respiratory or other medical condition limiting exercise tolerance
Fluency in English (verbal and written)	Any history of epilepsy
	LBP caused by involvement in a road traffic accident in the last 12 months or ongoing litigation
	History of serious Psychological or Psychiatric illness (mild depression eligible for inclusion)
	Current Pregnancy
	High activity levels categorised according to objective physical activity levels (7 day ActivPAL recording)

### Baseline outcome measurements

Baseline recording of all outcome measures shall be carried out by the same researcher prior to randomisation.

### Outcome Measures

#### Primary outcome

##### Functional disability

This will be assessed using the Oswestry Disability Questionnaire (ODQ). This has been shown to be a valid and reliable measure of pain and physical function in LBP patients [21]. The ODQ consists of 10 sections, each with six levels (with a maximum score in each section of five points) that assess the individual's limitations in various activities of daily living. The sum of all 10 sections is divided by the total possible score and the result multiplied by 100 to generate a percentage score. Values range from 0 (best health state) to 100 (worst health state) with an average score of 43% identified for chronic back pain participants [21-23]. A minimum important change of between 10-12 points over time, or an improvement from baseline of between 20-30% for an individual patient has been recommended [24].

#### Secondary outcomes

##### PA Level

Objective change in PA levels will be measured using an activPAL™ professional physical activity logger (PAL Technologies, Glasgow, UK). The activPAL is a small device (5 × 3 cm) containing a uni-axal accelerometer which provides a valid and reliable measure of steps taken, cadence and time spent lying/sitting, standing and stepping under free-living conditions. Reported ICCs for inter-device reliability range from 0.79-0.97 (CIs not stated) [25]. The monitor will be attached to the participant's anterior thigh using PALstickies™ and reinforced with Vulcan fixation tape (Mobilis Healthcare Group Limited, Oldham, Lancashire, UK). The device will be worn for seven consecutive days at each timepoint (baseline, eight weeks and 24 weeks). Each participant will also complete the IPAQ (short form) at each time point [26]. The IPAQ asks the participant about the time they spent being physically active in the last seven days.

##### Stage of Change

Readiness to change will be assessed using a standardised questionnaire, which describes an individual's position in a cycle of change described within the Prochaska and Diclemente framework [27].

##### Fear-Avoidance Beliefs - Physical Activity

Assessed using the Fear-Avoidance Beliefs Questionnaire (FABQ): The FABQ is a 16 item self-report questionnaire that specifically focuses on participants' beliefs about how PA and work affect their low back pain [28]. Only the PA items will be recorded. This method has previously been employed in other CLBP studies [20,29].

##### Back Beliefs questionnaire (BBQ)

The primary objective of the BBQ is to assess the individual's beliefs about various aspects of the future as a consequence of LBP. The scale comprises nine inevitability statements, along with five statements used as distracters. The scale is calculated by reversing and summing the 9 inevitability measure scores. The scale has been shown to have good internal validity (Chronbach's Alpha = 0.70) and reliability (Intraclass Correlation Coefficient = 0.87) [30].

##### Physical Activity Self-efficacy Scale

Self-efficacy was assessed using the five point scale proposed by Marcus et al [31]. Perceived self-efficacy is described as the belief or the confidence in owns own ability to perform a behaviour necessary to reach a desired goal or achieve an expected outcome[32]. Self efficacy is regarded as an important factor in the self-management of chronic conditions and is highly correlated with disability [33].

##### Pain

Pain will be assessed using a numerical rating scale (0-10). Participants will be instructed to select a number between 0 and 10 that best describes their pain over the last seven days with 0 meaning 'No pain' and 10 meaning the 'Worst pain imaginable' [24].

##### Health-related quality of life

This will be measured using the EuroQol-5D [34], a self-administered questionnaire that assesses the participant's health-related quality of life using a core set of five health-related quality of life items [35]. Its validity and reliability are supported, and it has been recommended for use in low back pain research [36]. For the UK population, an average weighted health index of 0.86 and self-rated health status of 82.48 have been reported in the literature [37]. Use of this outcome along with the information collected on use of health care resources will facilitate a cost-utility analysis of the trial interventions.

##### Health Care Usage

This will be assessed using a questionnaire developed by members of the research team in conjunction with a Health Economist. This tool has successfully been employed during a previous study [20].

Global Rating of change for physical activity [38]. At baseline participants will be asked to rate their ability to be more physically active compared to the previous week. This will be recorded as No change, Worse or Better. If they answer Worse or Better, they will be asked to indicate how much it is worse or better using one of the following markers: A tiny bit - almost the same; A little bit; Somewhat; Moderately; Quite a bit, A great deal or A very great deal. They will also be asked to rate how important this change or lack of change is to them using the same markers. At each follow up assessment, participants will be asked to give a rating compared to the last time point.

Prior to randomization, each participant's preference for which treatment they would like to receive will be recorded. Expectations of assigned treatment will also be recorded using a Likert Scale [20]. On completion of the trial a participant satisfaction questionnaire will be administered to ascertain views on the interventions and perception of benefit [20].

### Treatment allocation

A statistician who will have no contact with the day to day running of the study shall carry out all aspects of preparation for group randomisation. A randomisation sequence will be generated using computer software with an allocation ratio of 1:2. This is order to ensure more information can be gathered on the walking intervention group (Group B). This is relevant since we wish to gather as much detail as possible regarding adverse events or other side effects and will enable us to learn more about the type and level of training required for this intervention in a fully powered trial. Blank cards will be printed with Group A (Education and advice) or Group B (Education and advice plus a structured walking programme). These cards will be placed inside sequentially numbered opaque sealed envelopes. Following baseline assessment, these envelopes will be used to assign participants in sequence to one of the two treatment groups (Group A or Group B). Participants will therefore be randomised under strict double-blind conditions.

### Treatment protocols

#### Education & advice (Group A)

Participants in the Education and advice group will receive a single, one to one session with a physiotherapist who will complete a physical examination and give standardised advice using the 'Back Book' [17]. The aim of the session will be to encourage a graded return to normal activities. Specifically the session will address the causes of low back pain, as well as giving advice on how to deal with an attack of back pain, the nature of chronic pain and how to better cope with these issues. All participants will be given a copy of the Back Book to keep. This will have a contact telephone number for the physiotherapist printed on the inside of the front cover. Participants will be encouraged to contact the physiotherapist to seek advice or address any further questions that they may have at a later date during the intervention period. If they wish the participants can leave a message for the physiotherapist who will call them back using a standardised telephone script adapted from a previous study [20].

#### Education & advice plus a structured walking programme (Group B)

Participants in Group B will receive the same single education and advice session as described above for group A. In addition, they will also receive a graded pedometer-driven walking programme prescribed by the physiotherapist. Each participant will be given a pedometer (Yamax Digiwalker CW-701, Yamax, Japan) to monitor their activity. This has been shown to be the most reliable, commercially obtainable pedometer which is currently available [39]. Participants will have the pedometer for a one week familiarisation period before they return for their next appointment. At this appointment the specific content of the tailored intervention will begin. This will be determined according to the 5A's model of health behaviour advice. This model was developed for brief smoking cessation advice but has also been proposed as a tool by which clinicians working in a primary care setting might provide advice aimed at influencing other health related behaviours, including PA [40]. The 5A's are as follows: Ask, Assess willingness to change, Advise, Assist, and Arrange follow-up. During the study the model will therefore be applied in treatment group B according to the following format.

**1) Ask**: Participants will be asked about their current PA and exercise history. **2) Assess**: Participants current level of motivation to exercise will be measured using the readiness to change questionnaire which will have been recorded at baseline. Individuals in pre-contemplation will be asked their reasons for taking part in the trial and their barriers to further activity. They will then be asked how they feel about their exercise levels and if they are willing or ready to try a bit more. **3) Advise**: Participants will be given further advice on the benefits of PA for their back pain. This will be delivered using personalised but non-judgmental language. This will be based on the participant's responses to question 1 and will make reference to the information in the 'Back Book'. **4) **Assist: Participants will be given additional advice which will include an explanation of the use of pedometers to set individual goals and how this may be used to maintain motivation. There will then be a 10 minute self-efficacy walk, during which the participant will wear the pedometer while the physiotherapist will discuss aspects of walking as a form of PA (such as footwear and safety) as well as reinforcing some of the previously discussed aspects of the intervention. This will be followed by a brief discussion during which an initial, weekly step goal will be determined by negotiation. This will be set using the Goal Attainment Scale (GAS). This is order to place the step goal in a context which relates to the participants own circumstances. Participants will be asked to rate their perception of the likelihood of achieving the weekly goal on a scale of 0-10. If they rate the scale as below 8, the target will then be re-negotiated in order to reach a target which is seen as realistic by both the physiotherapist and participant. Lastly, the physiotherapist will demonstrate how to record daily step counts and any adverse events in a diary. This will also be used to determine compliance which will be based on the participants self-reported weekly step counts and recorded by the physiotherapist as a percentage of the target number of steps completed. **5) Arrange follow-up**: Appropriate follow-up and support will then be arranged. This will involve a discussion of a follow-up date or time frame to assess progress, to assist in overcoming difficulties and to reassess daily step goals. To encourage compliance, participants will receive a weekly telephone call from a physiotherapist using a pre-determined script (approximately 10-15 minutes). During this conversation, difficulties will be addressed, data from the pedometer recorded and a new walking goal set. This approach has been previously employed in other walking intervention studies [41]. In addition, participants will be encouraged to contact the physiotherapist if they have any other questions or concerns at any other time during the intervention period. The specific features of the five components will be recorded for each participant in a standardised manner.

### Follow-up procedure

Follow up measurements of all outcome measurements will be recorded at eight weeks (on completion of trial) and at six months after randomisation into the trial by a member of the research team who shall remain blinded to group allocation throughout the study.

### Focus Groups

Qualitative exploration of individuals' perception of walking will also be examined by use of focus groups at the end of the intervention. Participants in group B will be invited to one of three to five groups, consisting of between 5 and 8 participants, to discuss their experiences, expectations and satisfaction with the walking programme. Each focus group will consist of a maximum of eight participants and will take place over a two-hour period. A 'clue and cue process', using a checklist of topics, will be used to ensure that the same basic areas are covered, but allowing any issues of importance to emerge. Sessions will be moderated by an experienced, independent focus group moderator who will have had no previous contact with study participants prior to the focus group meetings. Audio-tape recordings and field notes will be prepared by another member of the research team not involved in the running of the trial. Interviews will be transcribed, and interpretation, synthesis and data reduction undertaken independently by two members of the research team. Analysis will then be undertaken using qualitative research and data analysis software (N-VIVO [QDSR]). The findings will be presented to participants and therapists for their feedback and to help inform further research.

### Sample size

No formal sample size calculation will be carried out. As a feasibility study, we aim to recruit a total sample of 50 participants (Approximately 17 in Group A and 33 in Group B according to the 1:2 randomisation methods). This sample size reflects both a realistic target for the intervention period and one which we anticipate will provide sufficient information on the interventions to inform future studies. In particular, we are interested in gathering as much information as possible on any adverse events (including minor musculoskeletal injuries) associated with the walking intervention. Two reported adverse events in the walking group will represent an approximate incidence of 5%.

### Statistical analysis

As a feasibility study, significance tests will not be performed or reported for the primary or secondary outcomes. Treatment effects will be represented by point estimates and confidence intervals. The assessment of participant satisfaction will be tabulated, as will adherence levels and any recorded difficulties or adverse events experienced by the participants or therapists. This information will be used to modify the planned interventions to be used in the main RCT. The following criteria would suggest that a main trial is not feasible: no apparent change in the outcomes with confidence intervals that include large negative values, feedback from participants that they were unable to complete (or lack of adherence with) the walking programme and/or unable to use the pedometers; high levels of musculoskeletal injuries occurring.

### Adverse event recording

Any adverse events will be reported using a standard proforma.

### Service user involvement

Two service users will sit on the research team panel during the developmental and running stages of the study.

### Training requirements

Both service users will attend two separate training sessions at the research centre to familiarise themselves with the study procedures. In addition, all physiotherapists involved in delivering the interventions will receive a standardised period of training prior to commencement of the study.

## Discussion

This paper describes the rationale and design of a study which will test the feasibility of using a structured, pedometer-driven walking programme in participants with CLBP. This study will be to provide important practical information on pedometer prescription for physiotherapists and people with CLBP. The longer-term objective is to provide the ground work necessary for a main trial to establish whether the use of such initiatives are more effective than simply giving advice to be more active.

## Competing interests

The authors declare that they have no competing interests.

## Authors' contributions

MT and SMcD conceived the idea for this study. SMcD, MT, DB, DH, AD, SON, and IB were involved in developing the original idea for funding and were co-applicants on the successful funding proposal. MT, CTL and SMcD devised a detailed walking programme. All authors participated in development of research protocols and design of the study. All authors read and approved the final manuscript.

## Pre-publication history

The pre-publication history for this paper can be accessed here:

http://www.biomedcentral.com/1471-2474/11/163/prepub

## Supplementary Material

Additional file 1 **Participant flow diagram**. This file describes the 3 recruitment routes and the interventions received in each group. Click here for file
