# Supplementary material for: The Back 2 Activity Trial: education and advice versus education and advice plus a structured walking programme for chronic low back pain
Source: BMC Musculoskelet Disord. 2010 Jul 15;11:163. doi: 10.1186/1471-2474-11-163 (PMC2914699; doi:10.1186/1471-2474-11-163)
Supplement: Additional file 1 — Participant flow diagram. This file describes the 3 recruitment routes and the interventions received in each group. [file 1471-2474-11-163-S1.DOC]

**Additional File 1: Participant flow diagram.** This file describes the 3 recruitment routes and the interventions received in each group.

**I:** Potential participant identified via physiotherapy waiting list

**II:** Potential participant identified from General Practice (GP) by PCRN

**III:** Potential participant identified via Occupational Health/e-mail or poster ad.

**T0**

Invitation letter sent

GP cover letter plus invitation letter sent

Invitation letter sent

Potential participant phones physiotherapy department

Potential participant returns reply slip

Potential participant contacts research team

Participants recruited via route I undergo formal screening procedures and interventions in physiotherapy department

Participants recruited via route II and route III undergo formal screening procedures and interventions in research centre

**▪**

**Appointment 1:** Full verbal explanation of study and opportunity to ask questions given to potential participant. Formal screening procedure carried out to confirm eligibility. Ineligible participants receive copy of the ‘Back Book’. Informed written consent gained from eligible participants and baseline outcome measurements recorded. Explanation and fitting of physical activity monitor (Physical activity levels recorded for 7 days). Appointment 2 confirmed.

**T1:**

**Week 1**

**Appointment 2:** Activity monitor removed and data checked to ensure final inclusion criteria met (required to be taking an average of less than 8,500 steps per day). All participants receive an education and advice session with the physiotherapist. Eligible participants then randomised under strict double blind conditions to treatment group A or B (8 week pedometer driven walking programme).

**T2:**

**Week 2**

**Group A (Controls)**

No further active intervention.

Participants may use telephone number to contact physiotherapist if they have any problems or questions

**Group B (8 week walking programme).**

Participants receive pedometer and explanation of use for 1 week familiarisation period [No initial step target given]. Appointment 3 confirmed.

**T3:**

**Week 3-10**

**Appointment 3 [Group B only]**

Participants receive session based on 5As plus self efficacy walk and first weekly step target [partly determined on basis of pedometer step count from familiarisation period]. Weekly telephone calls

from the physiotherapist with step counts reported and adjusted as required

**Follow-up assessment I** (8 weeks post randomisation)

Re-assessment of all outcomes (blinded) plus 7 day physical activity monitoring

**T4:**

**Week 10**

**Follow-up assessment II** (6 months post randomisation)

Re-assessment of all outcomes (blinded) plus 7 day physical activity monitoring

**T5:**

**Week 24**
